# The healing effects of thymoquinone on experimentally induced traumatic tendinopathy in rabbits

**DOI:** 10.1186/s13018-023-03706-8

**Published:** 2023-03-23

**Authors:** Alireza Soltanfar, Abdolhamid Meimandi Parizi, Mohammad Foad-Noorbakhsh, Mansour Sayyari, Aida Iraji

**Affiliations:** 1 Division of Surgery, Department of Clinical Sciences, School of Veterinary Medicine, Shiraz University, Shiraz, Iran; 2 Division of Pharmacology and Toxicology, Department of Basic Sciences, School of Veterinary Medicine, Shiraz University, Shiraz, Iran; 3 Stem Cells Technology Research Center, Shiraz University of Medical Sciences, Shiraz, Iran; 4 Central Research Laboratory, Shiraz University of Medical Sciences, Shiraz, Iran; 5 Department of Pathobiology, School of Veterinary Medicine, Shiraz University, Shiraz, Iran

**Keywords:** Thymoquinone, Tendon healing, Tendinopathy, Rabbits, Biomechanical, Biochemical, Histopathology

## Abstract

**Objectives:**

Thymoquinone is a major bioactive compound present in the black seeds of the *Nigella sativa*. Tendon injuries are almost 50% of all musculoskeletal injuries. The recovery of tendon after surgery has become a significant challenge in orthopedics.

**Design:**

The purpose of this study was to investigate the healing effect of thymoquinone injections in 40 New Zealand rabbits tendon traumatic models.

**Materials and methods:**

Tendinopathy was induced by trauma using surgical forceps on the Achilles tendon. Animals were randomly divided into 4 groups: (1) normal saline injection (control), (2) DMSO injection, (3) thymoquinone 5% w/w injection, and (4) thymoquinone 10% w/w injection. Forty-two days after surgery, biochemical and histopathological evaluations were done, and biomechanical evaluation was conducted 70 days after surgery.

**Results:**

Breakpoint and yield points in treatment groups were significantly higher compared to control and DMSO groups. Hydroxyproline content in the 10% thymoquinone receiving group was higher than all groups. Edema and hemorrhage in the histopathological evaluation were significantly lower in the thymoquinone 10% and thymoquinone 5% receiving groups compared to control and DMSO groups. Collagen fibers, collagen fibers with fibrocytes, and collagen fibers with fibroblasts were significantly higher in the thymoquinone 10% and thymoquinone 5% receiving groups compared to control groups.

**Conclusions:**

Thymoquinone injection in the tendon in the concentration of 10% w/w is a simple and low-cost healing agent that could enhance mechanical and collagen synthesis in traumatic tendinopathy models in rabbit.

## Introduction

Tendon problems usually present chronically with symptoms of pain, decreased biochemical activity, and decreased exercise tolerance (1). Unfortunately, this disease is common in horses and reduces their athletic performance, and even causes the termination of the animals’ careers. The superficial digital flexor (SDF) tendon injury is more frequent in horses compared to the other tendons (2). 82% of injuries and diseases of horses are related to musculoskeletal problems, of which 46% of them are diseases and injuries to tendons and ligaments (3).

Overuse of tendons and mechanical stress can cause tendinopathy which has been estimated to account for 30–50% of all sports-related injuries. Achilles tendinopathy presented as the second highest injury incidence at 9.1–10.9%. The major factor that induces tendinopathy is the loading of a tendon with physical activities without adequate time for tendon recovery (4–8). Pathoetiology of tendinopathies is multifactorial, but it is due to the degeneration of type I collagen as the result of the up-regulation of matrix metalloproteinases (MMPs) (9). This change in structure causes the production of inflammatory cytokines that slow down the healing process (10). Also, the healing process in the horse tendon is slow due to the lack of vascularity in the area (11). Following tendon injury, the expression of the MMP-1 gene, which is related to collagenase production, increases, and the strength, flexibility, and mechanical properties of the tendon decrease (2). These changes eventually cause varying degrees of pain and lameness in the animal (12). After tendon repair, there is a possibility of re-injury to the same tendon, causing tendinopathy (13).

Based on several articles, inflammation is the main key pathogenesis of tendon injury in both humans and equines. In case of tendon injury, the animal is usually given rest for 6 months to a year, as any exercise will cause more tendon injury (2). Available treatments for tendinopathy are pharmacological treatments such as non-steroidal anti-inflammatory drugs (NSAIDs) or in severe cases, surgery. But usually, these types of treatments rarely help the animal recover and return to normal sports activity (14).

Although regenerative medicine has received a great deal of attention today, and many articles have been published in the field of cell therapies by mesenchymal stem cells (MSCs), platelet-rich plasma (PRP), and autologous proteins or cells (15), there is still a need to study more compounds, especially compounds with anti-inflammatory and collagen production activity.

There are a variety of plant compounds that have anti-inflammatory, analgesic, and stimulating collagen production effects. *Nigella sativa* (NS) is a medicinal plant, and thymoquinone, (TQ), as its main active chemical component is reported to have analgesic, diuretic, antihypertensive, antidiabetic, anticancer, immunomodulatory, anti-inflammatory, and antioxidant properties (16). Thymoquinone has hepatoprotective, antihypertensive, diuretics, digestive, anticancer, appetite stimulant, antidiarrheal, anti-inflammatory, nephroprotective, neuroprotective, analgesics, and antibacterial activities which can stimulate collagen production in injured tissue (16).

So far, the healing effects of thymoquinone on various musculoskeletal diseases have not been investigated, but limited research has been done on tendinopathy. Due to the anti-inflammatory properties and collagen production of thymoquinone, in this article, we have investigated the healing effects of thymoquinone on tendinopathy caused in the Achilles tendon of rabbits. Histopathology, biomechanical and biochemical evaluation were used to evaluate its healing effect on tendinopathy.

## Materials and methods

### Materials

Thymoquinone was purchased from Sigma-Aldrich (CAS number: 490-91-5); hydroxy-proline kit was purchased from Kiazist (Kiazist Co., Hamedan, Iran). Formaldehyde solution was purchased from Merck; cryotube was purchased from Bio Plas Inc., xylazine and ketamine were purchased from Nasr (Iran), and rabbits were purchased from the animal center of Shiraz University of Medical Science.

### Preparation of thymoquinone solution

Two different concentrations of thymoquinone were prepared to identify the best concentration for tendinopathy healing. For this purpose, thymoquinone was diluted in DMSO + distilled water to obtain 5% and 10% w/w solution.

### Study design and animal model

A total of 40 New Zealand rabbits with an average body weight of 2.25 kg were used to induce tendinopathy in the Achilles tendon with the approval of the Animal Ethical and Welfare Committee of Shiraz Veterinary Medicine University (grant number: 10171). Ethical approval was confirmed by the Animal Care Committee of Shiraz University (IR. REC ethical code: 10171). The authors followed up all institutional and international guidelines for animal care and use during this study. The Animal Research Reporting in Vivo Experiments guidelines (ARRIVE) were also followed up. Each rabbit was kept in an individual laboratory rabbit cage. Before the beginning of the experiment, rabbits were housed for two weeks to adapt to the environment. The animals were maintained under controlled conditions of 25 °C ± 1 and 12-h light–dark cycles and had free access to a standard chow diet and water throughout the study. All rabbits were anesthetized with ketamine-xylazine (ketamine 30 mg/kg and xylazine 6 mg/kg, IM).

**Fig. 1:**
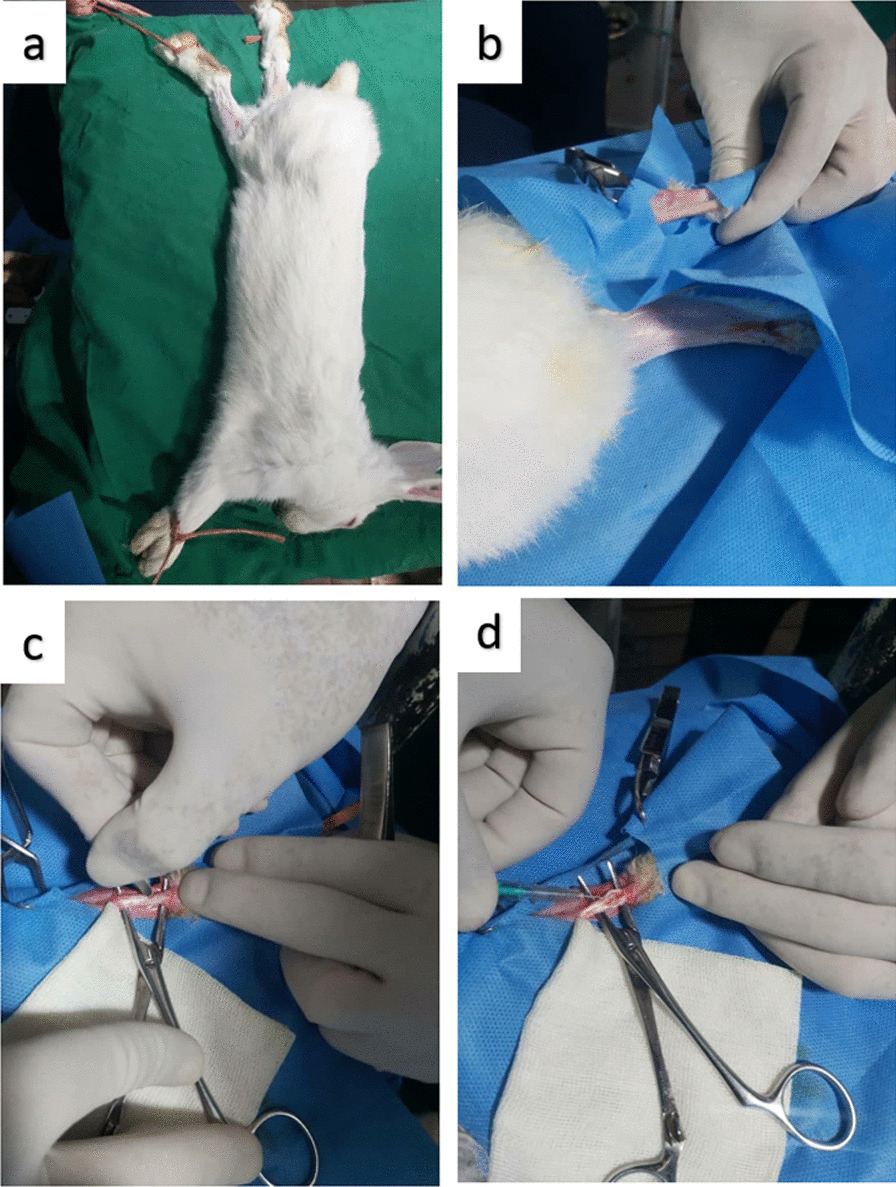
Surgical preparation and induced tendinopathy surgery in the rabbit

The left hind limb of each animal was used for tendinopathy induction. First, the area of Achilles' tendons was identified, and then, a longitudinal skin incision was made over the tendon. The paratenon was identified and incised longitudinally as a separate layer to expose the Achilles tendon. Hemostatic forceps were placed on the tendon for 60 s. After forceps removal, the treatment protocol was done as mentioned below (Fig. 1):

Group 1: Tendinopathy was induced and intratendinous injection was followed by normal saline (0.2 ml). Also, 0.2 ml normal saline was injected 3 days after surgery (*n* = 10).

Group 2: Tendinopathy was induced and intratendinous injection was followed by DMSO (0.2 ml). Also, 0.2 ml DMSO was injected 3 days after surgery (*n* = 10).

Group 3 (TQ 5): Tendinopathy was induced and intratendinous injection was followed by 5% w/w thymoquinone solution. Also, 0.2 ml 5% w/w thymoquinone was injected 3 days after surgery (*n* = 10).

Group 4 (TQ 10): Tendinopathy induced and intratendinous injection was followed by 10% w/w thymoquinone solution. Also, 0.2 ml 10% w/w thymoquinone solution was injected 3 days after surgery (*n* = 10).

In each group for the second time under general anesthesia, the incision site was opened again and 0.2 ml substance was injected into the tendon.

After surgery and full recovery, animals were returned to individual cages. Enrofloxacin (5 mg/kg, IM) was administrated to rabbits after surgery and continued for three days. All animals were clinically evaluated every week until the end of the sixth week, and lameness, swelling, and tenderness were assessed. Animals that lost weight a few days after surgery were excluded from the study and replaced with new animals.

### Biomechanical evaluation

The tensile test was performed in the biomechanics Department of the Faculty of Agriculture of Shiraz University. At the end of the experiment, five rabbits in each group were euthanized and the tendon was obtained. Biomechanical testing was done according to the methods of Oryan et al. (2012). After preparing the samples, they were wrapped with foil and stored at − 80 °C. The specimens were defrosted before the biomechanical examination. The samples were placed between two jaws of a tensile testing machine (Santam STM20) and stretched at a speed of 10 mm/min. Force–displacement graphs were drawn by the computer, and other information was obtained. The yield point and break point were read from the force–displacement curve to calculate yield stress and break stress. Also, the large and small diameter of the tendon at the lesion site was measured with a caliper (CALIPER) with an accuracy of 0.01 and calculated through the formula.

### Measurement of hydroxyproline

The amount of hydroxyproline in the tendon was measured using a hydroxyproline measuring kit (Kiazist Co., Hamedan, Iran). The protocol was performed according to the manufacturer’s catalog.

The samples taken for the pathology were divided into two equal parts exactly of the lesion repair site: the right-side samples were used for pathology and the other similar halves (weighing 20 mg) were used for measuring hydroxyproline. Briefly, the tissue samples were first taken out of the freezer; 20 to 40 mg of the sample was cut and placed in a microtube, and then, 100 μl of deionized water was poured into the microtubes and homogenized by the homogenizer. Then, 100 μl of HCl (12 M) was added to the microtube and incubated at 120 °C for 3 to 4 h. The sample was placed at 90 °C to evaporate the liquids. Fifty microliters of assay buffer was poured into each microtube and mixed. Next, 30 mg of activated charcoal was added to each sample and stirred thoroughly. It was then centrifuged at 12000 g for 15 min. The supernatant was used for further analysis. Each well of the kit was filled with 20 ml of the supernatant and detection solutions, and the absorption rate of the wells at a wavelength of 540–560 nm was read by Spectrophotometry Unicam UV vis (Thermo scientific, USA), and the concentration of the samples was obtained in comparison with a standard curve.

### Histopathology evaluation

Five animals in each group were euthanized at the end of the sixth week, and tendon samples were taken for histopathology evaluations. Samples were placed in 10% neutral formalin-buffer solution for 24–48 h for fixation, and formalin was changed every 24 h. Automatic tissue processing was used to prepare the tissue (brand name of the device). In this device, tissue samples were first placed in containers with alcohol with ascending subtility degrees. This step helps to remove excess water from the tissue and also the extra intracellular reactions stop. Then, tissue samples were transferred to containers containing xylol solution for clarification and alcohol removal and were finally embedded in paraffin. Four-micron sections were taken and stained with Hematoxylin & Eosin and Masson’s trichrome staining. All slides were evaluated using a light microscope (Olympus, Japan) and photographed by a camera (Olympus, Japan) under 40×, 100×, 200× and 400× magnification.

In histopathological examination, the inflammation score, collagen filament, and congestion or bleeding were scored qualitatively (Table 1). The scoring system was done based on Stoll et al. (17), with a slight change in scoring grade and protocol.

**Table 1:** Tendon healing scoring system based on histopathological findings

Parameter		Score
Inflammation	Absent inflammatory cell	0
	1–2 inflammatory cells (mild)	1 ( +)
	Inflammatory cells with giant cell (moderate)	2(+ +)
Edema	No congestion	0
	Congestion (mild)	1 ( +)
	Congestion (moderate)	2(+ +)
Hemorrhage	No hemorrhage	0
	Mild hemorrhage	1 ( +)
	Moderate hemorrhage	2 (+ +)
Collagen fibers	Few thin collagen fibers	0
	Few thick collagen fibers	1 ( +)
	Mild thick collagen fibers	2 (+ +)
	Abundant thick collagen fibers	3 (+ + +)
Collagen fibers with fibroblasts	No fibroblasts	0
	Mild fibroblasts	1 ( +)
	Moderate fibroblasts	2 (+ +)
	Abundant fibroblasts	3 (+ + +)
Collagen fibers with fibrocytes	No fibrocytes	0
	Mild fibrocytes	1 ( +)
	Moderate fibrocytes	2 (+ +)
	Abundant fibrocytes	3 (+ + +)

### Statistical analysis

For the analysis of histopathological evaluation, a nonparametric Kruskal–Wallis test was used. Statistical analyses of biomechanical and biochemical evaluation were performed with one-way ANOVA and post hoc Tukey's tests by GraphPad Prism 8(version 9- GraphPad). Any *p*-value less than 0.05 was considered to be statistically significant.

## Results

### Biomechanical findings

Yield points at week 6 were significantly different in the treatment group (TQ 5) than control and DMSO groups (Table 1, *p* = 0.0048). Break points in both treatment groups (TQ 5 and TQ 10) were significantly higher than in control and DMSO groups (TQ 5: control, DMSO *p* = 0.0005, *p* = 0.001. TQ 10: control, DMSO *p* = 0.007, *p* = 0.016, respectively). Yield stress in both treatment groups (TQ 5 and TQ 10) was significantly higher than in control and DMSO groups (TQ 5: control, DMSO *p* = 0.0003, *p* = 0.0006. TQ 10: control, DMSO *p* = 0.0007, *p* = 0.0014, respectively). Break stress in TQ 10 group was significantly higher than control and DMSO group (*p* = 0.0097, *p* = 0.032, respectively). Break stress in TQ 5 group was significantly higher than the control group (*p* = 0.016) (Table 2).

**Table 2:** Biomechanical parameters of the tendon in all groups

Groups	Yield point (N)	Breakpoint (N)	Yield stress (Mpa)	Break stress (Mpa)		
Control	35.34 ± 22.82^a^	40.42 ± 16.35^c^	8.23 ± 1.80^e^	9.050 ± 3.84^ g^		
DMSO	30.75 ± 20.39^a^	43.8 ± 17.79^c^	8.4 ± 1.67^e^	11.90 ± 4.83^ g^		
TQ 5	110 ± 37.2^b^	121.9 ± 32.03 ^d^	28.80 ± 5.95^f^	32.00 ± 12.00^i^		
TQ 10	80.0 ± 30.19^ab^	101.0 ± 27.22 ^d^	27.01 ± 9.39^f^	33.86 ± 15.46^i^		

Results are expressed as mean with S.D. In each column, different alphabet shows statistical difference against each other in different groups

### Hydroxyproline content

**Fig. 2:**
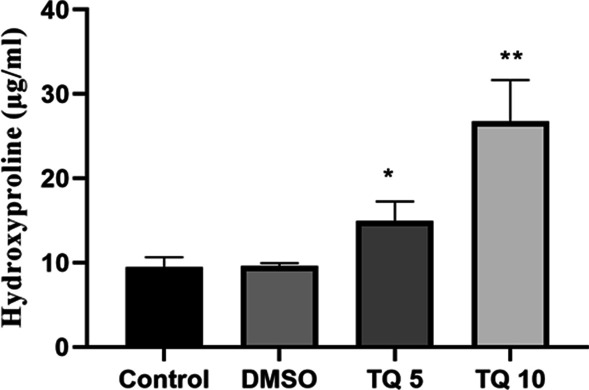
Hydroxyproline content (μg/ml) in tendons. Results are expressed as mean with S.E.M, and *p* < 0.05 is considered a significant difference. (*:*p* < 0.05 vs. control and DMSO, **: *p* < 0.01 vs. control and DMSO)

The hydroxyproline content per wet weight in TQ 5 was significantly higher than the control and DMSO groups (Fig. 2, *p* < 0.05). Also, 10% w/w thymoquinone solution could significantly increase the hydroxyproline content compared to the control and DMSO group with a *p* < 0.01.

### Histopathology findings

**Fig. 3:**
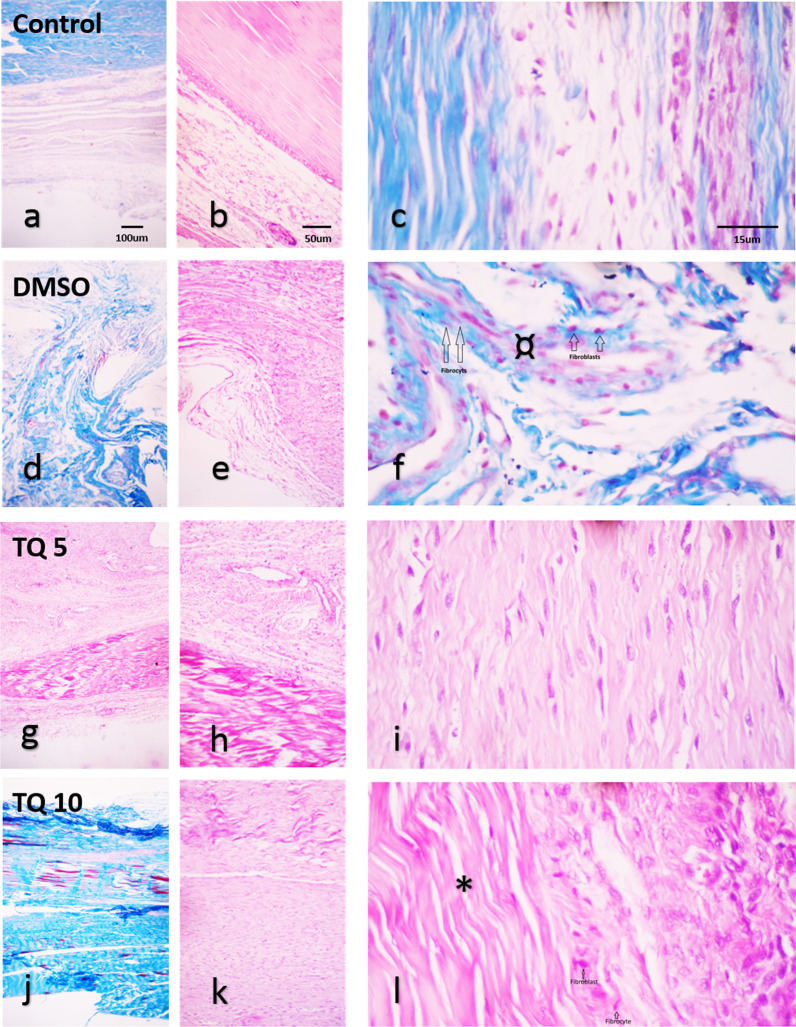
Histopathological image of all groups in 4, 10, and 40 × magnifications. H&E and Masson’s trichrome staining. * show dens and aligned collagen fiber and * shows unorganized collagen fiber in control and DMSO groups

**Fig. 4:**
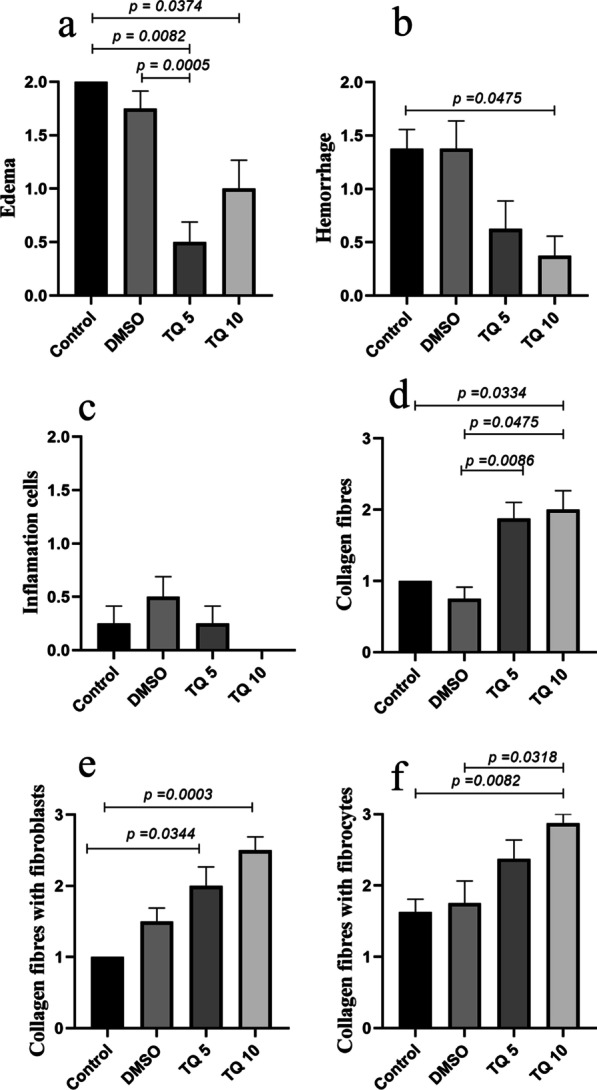
Histopathological parameters evaluation of tendons. Results are expressed as mean with S.E.M, and *p* < 0.05 is considered a significant difference

Six parameters were in histopathological evaluation. Three parameters consist of edema, inflammation, and hemorrhage (0–2 score), and three parameters consist of collagen fibers, collagen fibers with fibroblasts, and collagen fibers with fibrocytes (0–3 score). Histopathological sections of all groups are presented in Fig. 3. The number of fibroblasts and fibrocytes along the collagen fibers was higher in treatment groups than in control groups. In the control and DMSO groups, a cluster of round-shaped chondrocyte like cells were found at the repair zone (c,f) and collagen fibers were not organized in a regular orientation (b,d,e,f). In TQ 5 and TQ 10 groups, more density of collagen fibers that have aligned in a more orderly fashion was observed (i,l). Also, fibroblast migration increased in treatment groups than in the control and DMSO group. In edema parameters, there was a significant difference between treatment groups and control and DMSO groups (*p* < 0.05, Fig. 4a). Edema in TQ 5 group was significantly lower than control and DMSO groups. Edema in TQ 10 group was significantly lower than in the control group. There was no significant difference between the control and DMSO groups. In the hemorrhage parameter, there was a significant difference between TQ 10 and control groups (Fig. 4b). In inflammation cells, there was no significant difference between all groups (Fig. 4c). In the collagen fibers parameter, there was a significant difference between treatment groups and control and DMSO groups (Fig. 4d). Collagen fibers in TQ 10 and TQ 5 were significantly higher than DMSO group (Fig. 4e). Collagen fibers in the TQ5 group were significantly higher than in the control group. Collagen fibers with fibroblasts were significantly higher in TQ 10 and TQ 5 groups compared with the control group (Fig. 4e). Collagen fibers with fibrocytes were significantly higher in the TQ10 group than in the control group (Fig. 4f). Collagen fibers with fibrocytes were significantly higher in the TQ10 group than in the DMSO group (Fig. 4f).

## Discussion

Tendon injuries are almost 50% of all musculoskeletal injuries. Tendon recovery after surgery has become a significant challenge in orthopedics, so more investigation and better strategies for promoting tendon healing may provide new therapies for such patients. The purpose of this study was to investigate the healing effect of thymoquinone injections in rabbit tendon traumatic models. Although thymoquinone is well-known antioxidant, anti-inflammatory, and antibacterial effects (18), its injection effects on the traumatic tendon in rabbit models have not been studied yet. As a result, in the current study, the histopathological evaluation (H&E and mason trichrome), biomechanical, and biochemical evaluation was performed to investigate the healing effect of thymoquinone in the traumatic tendon in the rabbit model.

The tendon healing process consists of three major phases that overlap with each other: inflammation, formative, and remodeling phase (19). For optimized and rapid tendon healing, high production of collagen type I is necessary (19). In the current study, two-time injections three days apart of thymoquinone in the tendon demonstrated pronounced effects on collagen production and hydroxyproline content according to histopathological (Masson trichrome staining) and biochemical (hydroxyproline content) evaluations. In one study, the healing effect of polylactic acid/cellulose acetate along with thymoquinone on wounds was investigated. Their Masson’s trichrome staining showed significantly higher collagen deposition in the wound area (20). Many studies used thymoquinone as a hepatoprotective agent due to its upregulating effect on the AMP-activated protein kinase pathway (21, 22). Thymoquinone could increase the expression of the MAPKs, p-p38, p-ERK, p-JNK, and PI3K/pAkt to upregulate collagen I expression in a dose-dependent manner (23, 24).

The promising results of the current study could be due to not only the collagen production potential of thymoquinone but also due to the anti-inflammatory effects of thymoquinone that are certainly mentioned in many studies (25, 26). Thymoquinone inhibits tumor necrosis factor-α, interleukin-1B, and IL-6 production. These cytokines decrease collagen synthesis and activate MMPs that degrade collagen (27, 28). The canonical Wnt–wingless signaling pathway is well known and regulates many biologic processes by increasing the transcriptional activity and stability of β-catenin. Additionally, the Wnt pathway is important for wound healing because its key mediator β-catenin has a pivotal role in the proliferation phase of wound healing. β-catenin also participates in some phases of wound repair. First, phosphorylation occurs and it accumulates in the cytoplasm and then migrates into the nucleus. In the nucleus, the regulation of the target gene transcription occurs and this results in proliferation, migration, and accumulation in the collagen of fibroblasts (29–31). Pekmez and his colleagues showed that thymoquinone treatment increased β-catenin expression (32) which can result in proliferation, migration, and accumulation in the collagen of fibroblasts. Another reason for increased collagen and hydroxyproline content is due to the antioxidant activity of thymoquinone which could protect fibroblast from reactive oxygen species. Collagen type I is mainly produced by fibroblasts that replace the temporary fibrin-based matrix (33). A study conducted by Rahmani-Moghadam et al. used thymoquinone and hydroxyapatite to investigate its osteogenesis and collagen synthesis on mesenchymal stem cells. A real-time-PCR study demonstrated that thymoquinone-treated MSCs expressed collagen type I at the early phase of the differentiation phase (34).

One of the major problems in tendinous and ligamentous injuries in larger animals like the equine is the moderate prognosis of the horse to return to their racing activity. Treatment carried only a moderate prognosis for return to racing (62%), with a moderate rate of reinjury (46%) after treatment with growth factors (35). In rare cases, the tendon achieves functionality equal to that of the pre-injured state. Mostly, tensile strength is reduced up to 30% after injury (Müller et al., 2015). Tendons are related to the flexion of toes and foot movement, and to maintain this flexor movement, it is important to keep it in normal mechanical properties such as tensile load and normal diameter (36). Tendon healing after surgical repair generally progresses through a short inflammatory phase, which lasts about a week, followed by a proliferative phase, which lasts a few weeks, followed by a remodeling phase, which lasts many months (37). During the inflammatory phase, vascular permeability increases and an influx of inflammatory cells enters the healing site. These cells produce several cytokines and growth factors that lead to the recruitment and proliferation of macrophages and resident tendon fibroblasts. During the proliferative and remodeling phases of healing, fibroblasts proliferate and begin to produce, deposit, orient, and crosslink fibrillar collagens. Tendon is a dense connective tissue composed of highly organized parallel and longitudinal collagen fiber bundles (20, 38). The major ECM component in tendon tissues is type I collagen. Collagen contains at least one domain of repeated sequences of glycine (Gly)–X–Y, where X and Y are most frequently proline (Pro) and 4-hydroxyproline (Hyp), respectively (39). Commonly collagen content in connective tissues has been estimated by measuring the hydroxyproline content and assuming that it is present in collagen in a specific proportion based on the amino acid composition of collagen. The determination of Hydroxyproline content is in proportion to collagen content (40). Also, in 2015, Allahverdi et al. used the measurement of hydroxyproline in the evaluation of tendon repair (41). In another research, Jiang et al. used quantification of hydroxyproline for tendon repair evaluation (42). And also, Abd Al-Hussein et al. used the measurement of collagen content in the evaluation of tendon repair (43).

In our study, biomechanical evaluation showed an increase in yield point and break point in both treatment groups than DMSO and control groups. Biomechanical results are completely consistent with the hydroxyproline and histopathology results of our study. An increase in mechanical properties is due to the increase in collagen content, which has also been shown in our results. Based on histopathological results, collagen with fibrocyte and fibroblast was significantly higher in treatment groups, especially TQ 10 group than in control and DMSO groups. Rahmani–Moghadam study showed thymoquinone in the vicinity of mesenchymal stem cells induces a change in morphology after the second passage and cell morphology gradually changed to fibroblast-like cells (34). Another important thing that should be noticed is the type of collagen distribution in tendon healing. In early tendon healing, type III collagen is mainly present throughout the tendon tissue, but normal tendon contains type I collagen (19). Usually, after 40 days fibroblasts resorb collagen and produce new collagen with a more longitudinal orientation (19). In our study in a group treated with 10% w/w thymoquinone, more orientated fibers were seen in histopathological slides. So thymoquinone in a concentrate of 10% w/w could enhance fibroblast and fibrocyte proliferation and subsequently enhance collagen production as well as improve the remodeling phase by better fiber orientation in tendon healing.

This study evaluates the short-term effect of thymoquinone on rabbit Achilles tendon healing. A long-term investigation is needed to assess long-term biomechanical evaluation and also re-injury rate in rabbits.

## Conclusion

In conclusion, the promising biological results of thymoquinone injection in the Achilles tendinopathy model suggest this natural product as an ideal non-invasive initial treatment after tendon injury. Histopathological evaluation showed more fibroblast and fibrocyte proliferation and also improved fiber orientation after 42 days of tendon injury. Therefore, thymoquinone intratendon injection can be a low-cost and useful therapy for tendon injury.

## Abbreviations

TQ: Thymoquinone
GAGs: Glycosaminoglycans
MMP-1: Matrix metalloproteinase-1
NSAID: Non-steroidal anti-inflammatory drugs
PRP: Platelet-rich plasma
MSCs: Mesenchymal stem cells
NS: *Nigella sativa*
DMSO: Dimethyl sulfoxide
